# C‐terminal Cysteines of CueR Act as Auxiliary Metal Site Ligands upon Hg^II^ Binding—A Mechanism To Prevent Transcriptional Activation by Divalent Metal Ions?

**DOI:** 10.1002/chem.201902940

**Published:** 2019-10-15

**Authors:** Ria K. Balogh, Béla Gyurcsik, Éva Hunyadi‐Gulyás, Juliana Schell, Peter W. Thulstrup, Lars Hemmingsen, Attila Jancsó

**Affiliations:** ^1^ Department of Inorganic and Analytical Chemistry University of Szeged Dóm tér 7 6720 Szeged Hungary; ^2^ Laboratory of Proteomics Research Institute of Biochemistry, Biological Research Centre of the Hungarian Academy of Sciences Temesvári krt. 62 6726 Szeged Hungary; ^3^ Institute for Materials Science and Center for Nanointegration Duisburg-Essen (CENIDE) University of Duisburg-Essen, 45141 Essen (Germany), European Organization for Nuclear Research (CERN) 1211 Geneva Switzerland; ^4^ Department of Chemistry University of Copenhagen Universitetsparken 5 2100 Copenhagen Denmark

**Keywords:** coordination modes, CueR metalloregulatory protein, mercury, metal ion selectivity, perturbed angular correlation (PAC) spectroscopy

## Abstract

Intracellular Cu^I^ is controlled by the transcriptional regulator CueR, which effectively discriminates between monovalent and divalent metal ions. It is intriguing that Hg^II^ does not activate transcription, as bis‐thiolate metal sites exhibit high affinity for Hg^II^. Here the binding of Hg^II^ to CueR and a truncated variant, ΔC7‐CueR, without the last 7 amino acids at the C‐terminus including a conserved CCHH motif is explored. ESI‐MS demonstrates that up to two Hg^II^ bind to CueR, while ΔC7‐CueR accommodates only one Hg^II^. ^199m^Hg PAC and UV absorption spectroscopy indicate HgS_2_ structure at both the functional and the CCHH metal site. However, at sub‐equimolar concentrations of Hg^II^ at pH 8.0, the metal binding site displays an equilibrium between HgS_2_ and HgS_3_, involving cysteines from both sites. We hypothesize that the C‐terminal CCHH motif provides auxiliary ligands that coordinate to Hg^II^ and thereby prevents activation of transcription.

## Graphical abstract

**Intracellular Cu^I^ is controlled by the transcriptional regulator CueR**, which effectively discriminates between monovalent and divalent metal ions. Here, the binding of Hg^II^ to CueR and a truncated variant, ΔC7‐CueR without the last seven amino acids at the C‐terminus including a conserved CCHH motif, is investigated.

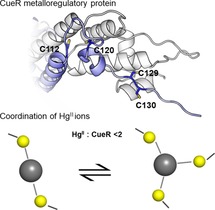

The CueR metalloregulatory protein controls cellular copper homeostasis by activating the transcription of *cueO* and *copA* genes in prokaryotes and some eukaryotes.^1^ CueR responds to Cu^I^, Ag^I^ and Au^I^, but not to the divalent ions Hg^II^ or Zn^II^.^2^ SC‐XRD studies on *Escherichia coli* CueR and EXAFS in solution revealed that the inducer metal ions are coordinated by C112 and C120 residues in a linear, bis‐cysteinate fashion.^2^, ^3^ These two cysteines are essential to the protein function, as shown by mutation studies (C112S and/or C120S) both in vitro^3^ and in vivo.^4^

CueR proteins from various bacteria contain two additional well conserved cysteines at the C‐terminal, disordered segment of the protein (Figure 1).^2^ Crystal structures of the activator and the repressor forms of the DNA‐bound CueR dimer suggest that a two‐turn helix between the metal binding loop and the CCHH motif may have a key role in the protein function.^5^ Upon Ag^I^ binding, the activator conformation is stabilized by the docking of the C‐terminal helix (via residues I122, I123, L126) into an opened, hydrophobic pocket, formed by residues of the dimerization helix and the DNA‐binding domain. This results in a small “scissoring” movement and bending of the DNA chain allowing the transcription to be carried out by the RNA polymerase. The allosteric role of the C‐terminal helix was confirmed by constructing the Cu^I^‐independent constitutive activator (T84V/N125L/C112S/C120S) and the constitutive repressor (truncation from I122) mutants of CueR.^5^

**Figure 1:**
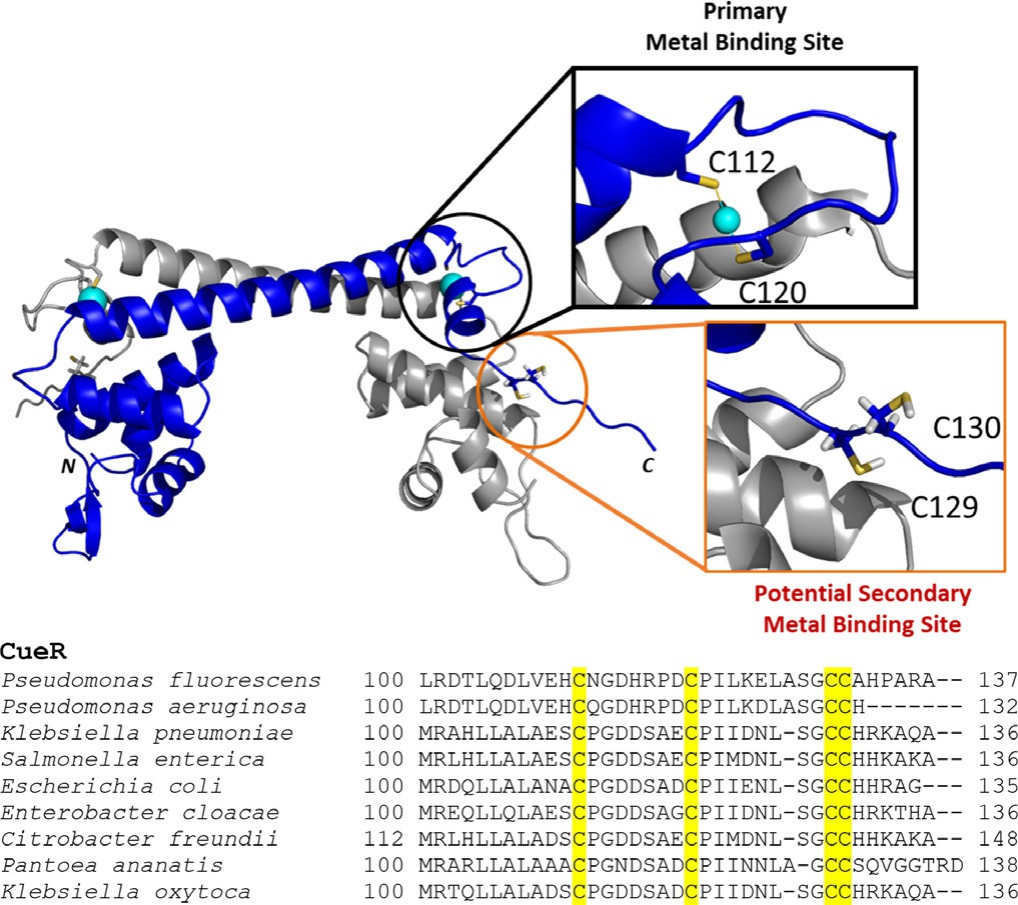
Structure of CueR (*E.coli*) (PDB id.: 1Q05‐modified) showing the potential metal binding sites (top). Sequence alignment of CueR proteins from various organisms (bottom). Conserved cysteine residues are highlighted in yellow.

Several representative examples can be found in the literature where non‐cognate metal ions bind to a metalloprotein with the same or even higher affinity than the inducer metal ion. However, despite the high affinity binding of non‐cognate metal ions, they cannot trigger the functional structural change of the protein, because the coordination number or geometry differ.^6^, ^7^, ^8^, ^9^ Thus, studying the interaction of metallo‐regulatory proteins with non‐cognate metal ions may provide a deeper insight into the mechanism of metal ion selection and the regulation of the transcription.^8^

Although CueR is one of the most thoroughly characterized proteins in the MerR family, the mechanism of discrimination between mono‐ and divalent metal ions is still not fully understood. Surprisingly, Hg^II^ does not trigger the activation of transcription by CueR,^2^ despite its well‐known preference for a bis‐thiolate coordination environment.^10^ O'Halloran et al. determined a Cu^I^‐binding sensitivity of the CueR protein (1–2×10^−21^ m) based on an in vitro transcriptional assay.^2^ Our previous studies on model peptides of the metal binding loop of CueR also showed that these fragments bind Cu^I^ with a high affinity.^11^ However, according to model peptide studies^12^, ^13^ and QM/MM calculations,^14^ Hg^II^ ions may be coordinated even more efficiently. Moreover, Hg^II^ is also able to bind to a CC sequence,^15^ and therefore coordination of Hg^II^ ion by the CCHH motif is also highly probable.

With the present work we aim to explore the role of the C‐terminal CCHH motif with a particular focus on the binding of Hg^II^ to CueR. To achieve this, we studied the Hg^II^‐interaction of *E. coli* CueR and its truncated variant, lacking seven C‐terminal residues (including the CCHH motif), ΔC7‐CueR. The integrity of this variant was confirmed by CD spectroscopy and electrophoretic mobility shift assay, see Figure S3.

A series of ESI‐MS spectra were recorded with the two protein variants, see Figures 2, S4 and S5. The disappearance of the signals of the apo‐form in the presence of 1.0 equivalent of Hg^II^ implies that Hg^II^ ions display high affinity to both proteins. The spectra obtained at twofold Hg^II^‐excess per protein clearly demonstrate the availability of two binding sites for Hg^II^ ions in the Wild‐type (WT) CueR. These are most likely the metal ion binding loop formed by C112 and C120, and the C‐terminal CCHH motif. Participation of the latter CCHH sequence motif in Hg^II^ binding is supported by the lack of signals corresponding to a Hg_2_‐ΔC7‐CueR complex, even at twofold Hg^II^‐excess over the truncated protein. Both the Hg‐CueR and Hg_2_‐CueR species are observed at 1.0 equivalent Hg^II^, suggesting that there is no significant difference in the Hg^II^‐binding affinities of the two sites.

**Figure 2:**
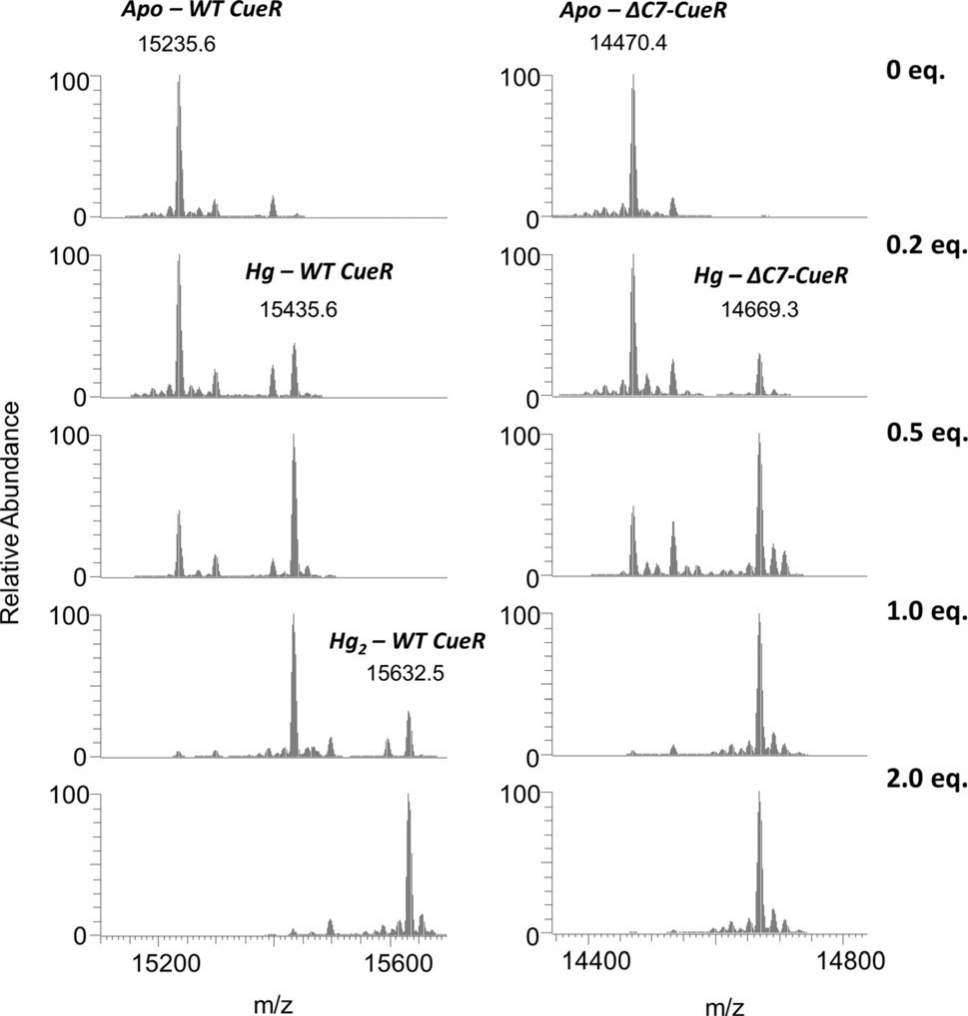
Deconvoluted native ESI‐MS spectra of the WT and truncated CueR in the absence and presence of 0.2, 0.5, 1.0 and 2.0 equivalents of Hg^II^ ions. Individual samples contained 20 μm protein in a 10 mm NH_4_HCO_3_ buffer, 0.5 mm TCEP, pH 7.5.

^199m^Hg‐perturbed angular correlation (PAC) spectroscopy^12^, ^13^, ^16^, ^17^, ^18^, ^19^, ^20^ was used to elucidate the metal site structures and dynamics at the nanosecond timescale, see Figure 3 and Supporting Information Figure S6. At pH 6.0 and Hg^II^:CueR of 0.2 and 1.0, the signals agree well with a HgS_2_ coordination geometry, that is, coordination of Hg^II^ by two cysteinates.^18^ This is also the case at Hg^II^:CueR of 2.0, although a slightly larger linewidth is observed, in particular for the first peak at around 1.4 rad ns^−1^. This line broadening presumably reflects the occupation of two HgS_2_ sites, and it can originate either from minor differences in structure of the two sites, or from metal site dynamics at the nanosecond time scale becoming more pronounced upon binding of the second Hg^II^ (Figure 3).

**Figure 3:**
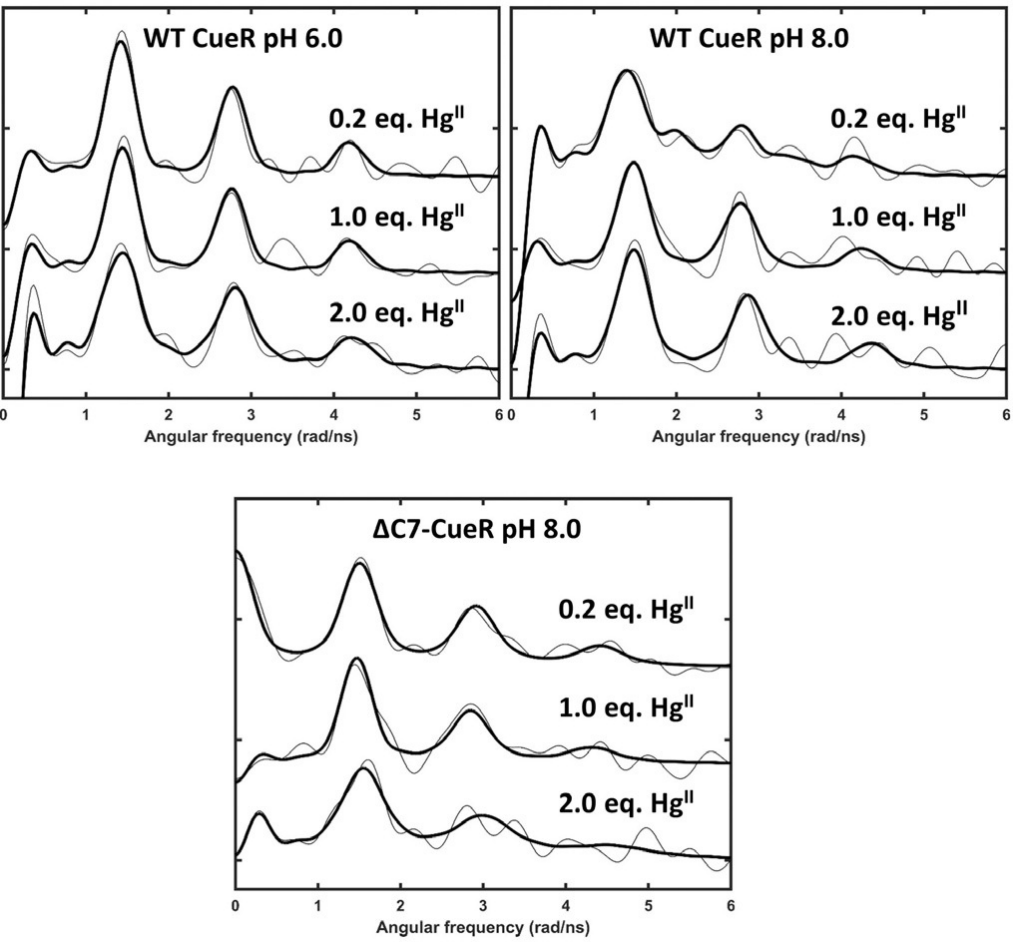
Experimental (grey) and fitted (black) ^199m^Hg PAC spectra of WT and truncated CueR in the presence of DNA with 0.2, 1.0 and 2.0 equivalents of Hg^II^. Top left: WT at pH 6.0; top right: WT at pH 8.0 *c*_WT CueR_=12 μm, 0.5 equiv. DNA, and bottom: ΔC7‐CueR at pH 8.0 *c*_ΔC7‐CueR_=8.4 μm, 0.5 equiv. DNA.

The spectrum recorded with 0.2 equivalent of Hg^II^ per CueR at pH 8.0 is more complex than at pH 6.0. Qualitatively, the first peak is shifted to slightly lower frequency and exhibits considerable broadening, and the second peak (ca. 2.8 rad ns^−1^) is significantly attenuated, to the extent that it barely rises above the noise level. A reliable analysis of the data requires the inclusion of two nuclear quadrupole interactions (NQIs). One of these NQIs is very similar to that observed in the spectra at pH 6.0, most likely reflecting a HgS_2_ structure. The other NQI has a higher asymmetry parameter and a lower frequency, see Table S1, indicating a higher coordination number than 2. The lower frequency agrees well with an ideal trigonal planar HgS_3_ structure, but the relatively high asymmetry parameter rules out this possibility. However, in the simple angular overlap model (AOM),^21^ a T‐shaped HgS_3_ coordination geometry gives the same frequency as a trigonal planar structure, but an asymmetry parameter of 1. Thus, a HgS_3_ structure in between trigonal planar and T‐shaped, with the third ligand in a slightly longer Hg−S distance seems to be a plausible structural interpretation of the low frequency signal. It is also possible that the PAC data reflect a trigonal planar HgS_2_N structure, with a histidine coordinating, as this would give an asymmetry parameter different from zero. However, this seems less likely, given the thiophilicity of Hg^II^, and the UV absorption data, vide infra. Finally, it is conceivable that the spectrum reflects intermediate (nanosecond) exchange between HgS_2_ and HgS_3_ structures. Notice that this entails a flip of principal axis of the electric field gradient tensor, which has *V*_zz_ along the axis of HgS_2_ but perpendicular to the HgS_3_ plane, and therefore the asymmetry parameter will depend on the dynamics in a non‐trivial manner. It cannot be excluded that the data recorded at 1.0 equivalent of Hg^II^ also contain signals reflecting both of these species, but the reduced chi‐square does not improve significantly upon including a second NQI. Consequently, we have only included the high frequency NQI (HgS_2_) in the analysis. For the experiment with 2.0 equivalents of Hg^II^ the signal may be satisfactorily fitted with just one (high frequency) NQI, presumably reflecting HgS_2_ structure for both Hg^II^ bound to CueR (Figure 3).

Most interestingly, the ^199m^Hg PAC spectrum recorded at pH 8.0 with 0.2 equivalents Hg^II^ for ΔC7‐CueR exhibits a signal reflecting only HgS_2_ structure (Figure 3). The fact that the ΔC7‐CueR Hg^II^ site exhibits a HgS_2_ structure strongly supports the interpretation presented above for the WT CueR: if HgS_3_ is formed by occupation of the functional site, a third thiolate is recruited from the CCHH motif, or vice versa, Hg^II^ binds to the CCHH motif and recruits one of the cysteines from the functional binding site. With 2.0 equivalents of Hg^II^ per ΔC7‐CueR at pH 8.0, the signal changes as compared to experiments with ≤1 equivalent Hg^II^, presumably because the functional metal site is filled, and the additional Hg^II^ accommodates a coordination geometry other than linear HgS_2_ due to weak or non‐specific Hg^II^ adducts. This agrees well with the ESI‐MS data, where no Hg_2_‐ΔC7‐CueR was observed. Thus it is likely that the signal includes more than one NQI. Surprisingly, the signal shifts to slightly higher frequency, which is difficult to account for, except if a positive charge appears in the equatorial plane of HgS_2_, vide infra.

To further characterize the metal site coordination geometries, we applied UV absorption spectroscopy (Figure 4). Hg^II^‐thiolate complexes possess characteristic charge transfer (CT) bands in the region of 230–300 nm. Moreover, features of the absorption spectrum reflect the coordination geometry of the complexes. Using Hg(SEt)_2_ and [Et_4_N][Hg(SBut)_3_] model compounds, the UV‐absorption spectra of linearly and trigonal planar coordinated Hg^II^, respectively, were characterized.^24^ Linearly coordinated Hg^II^‐thiolate species display a transition at around 230 nm.^22^ The increase of the coordination number shifts the absorption bands towards longer wavelengths.^23^, ^25^ The spectrum of a trigonal Hg^II^‐thiolate complex has a characteristic absorption maximum at 245 nm with a distinct shoulder at around 290 nm.^22^ Qualitatively, the absorption difference spectra at sub‐equimolar Hg^II^:WT CueR ratios exhibit a characteristic absorption at around 290 nm reflecting the presence of HgS_3_ structure (Figure 4), in agreement with the PAC data, vide supra. The PAC data indicate 40 % HgS_3_ and 60 % HgS_2_ at 0.2 equivalents Hg^II^. We used the recorded spectrum with 2.0 equivalents Hg^II^ per WT CueR (Figure 4 A) to determine the molar absorption of the HgS_2_ species (green curve in Figure 4 C). Next, we predicted the pure HgS_3_ molar absorption spectrum (Figure 4 C, purple curve) by assuming that the experimentally determined spectrum is given by 0.6 HgS_2_+0.4 HgS_3_. The UV absorption spectra derived in this manner for HgS_2_ and HgS_3_ agree well with those reported in the literature,^23^ strongly supporting the interpretation of the PAC data presented above. We present molar absorption data at selected wavelength values in Table 1. The UV absorption spectra recorded for ΔC7‐CueR exclusively exhibit the signature of HgS_2_ structure, corroborating the interpretation of other experimental data. Surprisingly, the absorbance for ΔC7‐CueR continues to increase beyond 1.0 equivalent Hg^II^ and saturates at ca. 2:1 Hg^II^:ΔC7‐CueR, indicating that the truncated protein can accommodate two Hg^II^ ions in a HgS_2_ coordination environment. This may be realized if a dinuclear Hg_2_S_2_ site is formed with the two thiolates as bridging ligands. Interestingly, this agrees with the unexpectedly high frequency observed by PAC spectroscopy, which can be explained by the presence of a positive charge (the second Hg^II^) in the Hg_2_S_2_ structure, vide supra. The fact that the species with two Hg^II^ bound per CueR monomer is not observed in ESI‐MS implies that the binding of the second Hg^II^ is relatively weak.

**Figure 4:**
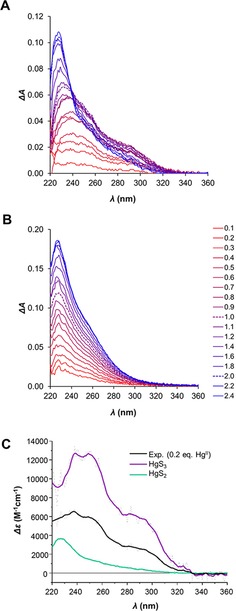
UV absorption difference spectra of WT CueR (**A**) and ΔC7‐CueR (**B**) titrated with Hg^II^ ions (0.1–2.4 equivalents). Spectra recorded in the presence of 1.0 and 2.0 equivalents of Hg^II^ are shown with dashed lines. pH 7.5, *c*_WT CueR_=14 μm, *c*_ΔC7‐CueR_=12 μm. (**C**) Estimated molar absorbance for the HgS_2_ and HgS_3_ species derived from the WT CueR UV absorption spectrum recorded with 2.0 equiv. Hg^II^ and 0.2 equiv. Hg^II^ combined with the relative population of the two species derived from ^199m^Hg PAC data, see the text for details.

**Table 1:** Spectroscopic properties of the HgS_2_ and HgS_3_ species compared to Hg^II^/MerR and Hg/L16C complexes. The two entries for CueR are from this work, see Figure 4 C.

Sample	*λ* [nm]	*ϵ* [cm^−1^ mm ^−1^]
Hg‐MerR^22^	242	19.8
	260	14.6
	290	6.45
		
1:2 Hg/L16C^23^	240	2.70
		
1:3 Hg/L16C^23^	247	16.8
	265	10.6
	295	5.00
		
CueR (HgS_3_)	240	12.6
	250	12.6
	290	5.82
		
CueR (HgS_2_)	230	3.52

In Figure 5, we present model structures which agree with all the experimental data presented in this work. At pH 8.0 with 0.2 and 1.0 equivalent Hg^II^, two species co‐exist, most likely the linear HgS_2_ and a HgS_3_ structure with the equatorial Hg‐S bond being longer than the other two. Such structures have also been observed in small, Hg^II^ containing inorganic compounds.^26^ The increased availability of deprotonated cysteines with increasing pH agrees well with this change in speciation observed from pH 6.0 to pH 8.0, that is, a change from HgS_2_ towards HgS_3_ coordination mode, and a similar trend has been observed for de novo designed proteins by Iranzo et al.^18^ The additional thiolate is most likely recruited from the CCHH motif, or vice versa, and may thus prevent the docking of the C‐terminal helix into the hydrophobic pocket, and consequently inhibit activation of transcription. The net negative charge of HgS_3_ may be stabilized due to the presence of lysine or arginine in the C‐terminal fragment of CueR in almost all the organisms listed in Figure 1. That is, we hypothesize that the CCHH motif is not involved in the function of CueR when sensing the monovalent coinage metals, but it does take part in binding of divalent metal ions, a mechanism that would account for the selectivity of CueR.

**Figure 5:**
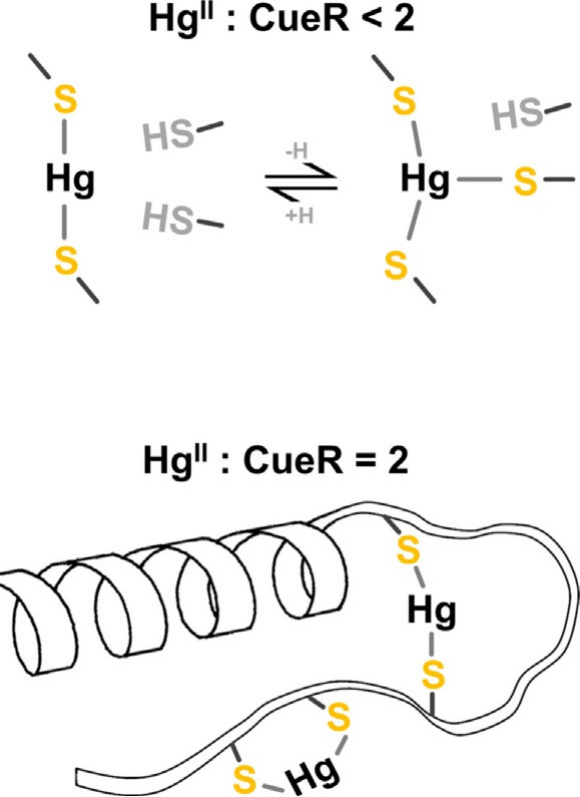
Model structures of Hg^II^ bound to WT CueR at pH 8.0. Binding of Hg^II^ to CueR gives rise to an equilibrium between HgS_2_ and HgS_3_ when Hg^II^:CueR <2, and to pure HgS_2_ coordination upon addition of 2 Hg^II^ ions per protein monomer. This can only be accounted for if the CCHH C‐terminal motif participates in the coordination of Hg^II^, see the text for details.

It may seem intriguing that with 1.0 equivalent Hg^II^ both the PAC and UV absorption spectra differ significantly from those recorded with 0.2 equivalent Hg^II^. However, a simple probabilistic model qualitatively accounts for this change, assuming that the two sites are independent (i.e. distributing Hg^II^ randomly among the 4 metal sites of a protein dimer), and that population of two adjacent sites (the functional site and the C‐terminal site) leads to formation of HgS_2_, because there are no more cysteines locally available to form HgS_3_, see the Supporting Information for details. This very simple interpretation is to some extent supported by the ESI‐MS data, which display population of the Hg_2_‐CueR species when Hg^II^ and CueR are present in equimolar amounts. Obviously, the model is too simple because formation of HgS_3_ requires that cysteines from both metal binding sites are involved, but the alternative, that is, that one binding site (either the functional site or the C‐terminal site) binds Hg^II^ with significantly higher affinity than the other, does not agree with the spectroscopic data, because this would imply that the HgS_2_/HgS_3_ ratio should be the same at 0.2 and at 1.0 equivalent, nor with the ESI‐MS data, which indicate the presence of Hg_2_‐CueR already at 1.0 equivalent Hg^II^. At 2.0 equivalents Hg^II^, of course, there is no more possibility to form HgS_3_, because the protein is saturated with Hg^II^ in HgS_2_ structures. Similar geometrical rearrangement was observed in metallothioneins (by UV absorption) upon saturating the protein by the metal ion in a titration with Hg^II^.^27^, ^28^ The function of the CCHH motif has also been studied by Stoyanov and Brown, using an in vivo assay to monitor the CueR controlled transcription.^4^ The double mutation of histidine (H131N/H132N) or cysteine residues (C129S/C130S) and truncation from G128 in *E. coli* CueR resulted in an only slightly altered induction of the transcription by cognate metal ions. Although experimental data were not presented, Stoyanov and Brown indicated that the selectivity of reaction with other, unspecified metal ions was not affected. To further explore this issue, a series of in vitro and in vivo transcriptional assays should be conducted.

In summary, we have demonstrated that up to two Hg^II^ ions bind with high affinity to WT CueR, one at the functional (C112 and C120) metal binding site, and the other at the C‐terminal CCHH motif. Moreover, under conditions where the protein is not saturated by Hg^II^, a higher coordination number (presumably HgS_3_) is observed for WT CueR but not for ΔC7‐CueR, indicating that side chains from the CCHH motif may be recruited as auxiliary ligands at the functional metal site (or vice versa). This implies a mechanism where the specificity of CueR for monovalent coinage metal ions and against divalent metal ions is achieved by coordination to divalent metal ions by the CCHH motif, preventing the docking of the C‐terminal helix into the hydrophobic pocket,^5^ and consequently inhibiting activation of transcription. Indeed, the CCHH motif provides a selection of ligands that may participate in coordination of both soft and intermediate metal ions. As the findings presented here on Hg^II^ do represent a special case, the generalization to other divalent metal ions should be considered carefully.

## Conflict of interest

The authors declare no conflict of interest.

## Supporting information

As a service to our authors and readers, this journal provides supporting information supplied by the authors. Such materials are peer reviewed and may be re‐organized for online delivery, but are not copy‐edited or typeset. Technical support issues arising from supporting information (other than missing files) should be addressed to the authors.

Supplementary
