# Supplementary material for: C‐terminal Cysteines of CueR Act as Auxiliary Metal Site Ligands upon HgII Binding—A Mechanism To Prevent Transcriptional Activation by Divalent Metal Ions?
Source: Chemistry. 2019 Oct 15;25(66):15030–5. doi: 10.1002/chem.201902940 (PMC6899792; doi:10.1002/chem.201902940)
Supplement: Supplementary file 1 — Supplementary [file CHEM-25-15030-s001.pdf]

CHEMISTRY
A European Journal

Supporting Information

C-terminal Cysteines of CueR Act as Auxiliary Metal Site Ligands upon Hg^{II} Binding—A Mechanism To Prevent Transcriptional Activation by Divalent Metal Ions?

Ria K. Balogh,[a] Béla Gyurcsik,[a] Éva Hunyadi-Gulyás,[b] Juliana Schell,[c] Peter W. Thulstrup,[d] Lars Hemmingsen,*[d] and Attila Jancsó*[a]

chem_201902940_sm_miscellaneous_information.pdf

Table of contents

Protein and DNA sequences 2

Cloning, purification and identification of proteins 2

Mass spectrometry 3

UV absorption spectroscopy 3

^{199m}Hg perturbed angular correlation spectroscopy 3

Quality control of the CueR proteins 4

^{199m}Hg PAC measurements with WT CueR in the presence of DNA 5

Model for the binding of Hg^{II} to the functional and *C*-terminal binding sites 5

Figures 7

Figure S1: SDS-PAGE analysis of the purified ΔC7-CueR protein. 7

Figure S2: Measured (A) and deconvoluted monoisotopic mass spectra (B) of the purified ΔC7-CueR protein. 7

Figure S3: (A) CD spectra of the Wild-type and ΔC7-CueR proteins ($c_{protein}$ = 25 μM) **(B)** EMSA analysis of ΔC7-CueR and 35 bp specific and non-specific DNA fragments. Lane *1* and *6* contain the 100 bp DNA ladder (GeneRuler, Thermo) for comparison. The protein-free specific and non-specific DNA samples are in lanes *2* and *4* respectively, while protein-DNA mixtures are in lanes *3* and *6*. 8

Figure S4: Comparison of the experimental and calculated isotopic envelop of WT CueR, Hg-WT CueR and Hg_2-WT CueR species (calculations were executed with Protein Prospector ver. 5.22.1 – UCSF). 9

Figure S5: Comparison of the experimental and calculated isotopic envelop of ΔC7-CueR and Hg-ΔC7-CueR species (calculations were executed with Protein Prospector ver. 5.22.1 – UCSF). 10

Figure S6: Experimental and fitted ^{199m}Hg PAC spectra of the WT CueR in the absence of DNA at pH = 8.0 with 0.2, 1.0 and 2.0 equivalents of Hg^{II}. $c_{WT\ CueR}$ = 11.5 μM. 11

Tables 12

References 13

Protein and DNA sequences

Wild-type (WT) CueR:
MNISDVAKITGLTSKAIRFYEEKGLVTPPMRSENGYRTYTQQHLNELTLLRQARQVG
FNLEESGELVNLFNDPQRHSADVKRRTLEKVAEIERHIEELQSMRDQLLALANACPG
DDSADCPIIENLSGCCHHRAG

ΔC7-CueR:
MNISDVAKITGLTSKAIRFYEEKGLVTPPMRSENGYRTYTQQHLNELTLLRQARQVG
FNLEESGELVNLFNDPQRHSADVKRRTLEKVAEIERHIEELQSMRDQLLALANACPG
DDSADCPIIENLSG − − − − − − −

P*copA* dsDNA: specific double strand DNA fragment including the recognition site of CueR protein *i.e.,* the promoter sequence of *copA* gene:

5' – AAAGGTTAAACCTTCCAGCAAGGGGAAGGTCAAGA - 3'

Cloning, purification and identification of proteins

The gene of *E. coli* CueR was kindly provided by prof. Alfonso Mondragon (Department of Molecular Biosciences, Northwestern University, Evanston, Illinois, USA).[1] Initially, the gene of CueR was recloned into pET-21a DNA vector (Novagen) as described previously.[2] The gene of *C*-terminally truncated CueR protein was constructed using a conventional restriction enzyme digestion/ligation cloning method. As a first step, the shortened gene was amplified from pET-21a-CueR plasmid with 5'-TAATACGACTCACTATAGGG-3' forward and 5'-TC<u>GGATCC</u>**TCA**GCCGGAGAGATTTTC-3' reverse primers in PCR (the underlined nucleotides in the reverse primer denote the BamHI recognition site, while the inverse of the bold triplet is the stop codon). The obtained DNA fragment was digested by NdeI and BamHI (Thermo Scientific) restriction enzymes and ligated by T7 Ligase (NEB) into the pET-21a plasmid digested with the same enzymes. *E. coli* Mach1 (Invitrogen) competent cells were transformed with the resulted plasmid for DNA multiplication. The new construct was sequenced to confirm the success of cloning.

The expression and purification procedures of the mutant CueR protein were performed as described previously for the Wild-type protein,[2] except that the second anion exchange step was eliminated. Protein samples were analysed by Tricine-SDS-PAGE[3] with Coomassie staining. The purified CueR variant was analysed by mass spectrometry. The intact molecular mass was measured from an acidified and desalted sample. The DNA binding capability was investigated with electrophoretic mobility shift assay. Both procedures were performed similarly like previously.[2]

Mass spectrometry

Mass spectrometric experiments were performed on an LTQ-Orbitrap Elite (Thermo Scientific) mass spectrometer coupled with a TriVersa NanoMate (Advion) chip-based electrospray ion source. The capillary voltage was 1.3 – 1.4 keV and the source temperature was 300 °C. We used Xcalibur 2.2 (Thermo) software for data evaluation and deconvolution.

Native mass spectra were measured from samples containing Wild-type or truncated CueR at a protein concentration of 20 μM (10 mM ammonium bicarbonate/NH_3, 0.5 mM TCEP, pH 7.5). 0.2, 0.5, 1.0 and 2.0 equivalents of Hg^{II} was added to individual samples from a $HgCl_2$ stock solution ($c_{Hg^{II}}$ = 250 μM).

UV absorption spectroscopy

UV absorbance spectra were recorded with a Shimadzu UV2600/UV3600 spectrophotometer in a wavelength range of 200-390 nm with 1 nm steps. The titration was carried out in a Hellma semi-micro quartz cell with 10 mm optical path length supplemented with magnetic stirrer and Teflon stopper. Initial concentration of the WT and ΔC7-CueR were 14 and 12 μM, respectively. Samples were buffered with Tris (20 mM Tris/$HClO_4$, 0.3 mM TCEP, pH 7.5). 7.0 or 6.0 μL aliquots (corresponding to 0.1 eq. Hg^{II} per CueR monomer) of a $HgCl_2$ stock solution ($c_{Hg^{II}}$ = 2.4 ×10^{-4} M) was added to the protein samples. From all the recorded spectra the spectrum of Tris buffer was subtracted as a baseline. The difference spectra were obtained by subtracting the spectra of apo-protein ($\Delta A = A_{\text{Hg-protein}} - A_{\text{apo-protein}}$) and the offset of the baseline (*i.e.* the absorbance measured at 390 nm).

Circular dichroism spectroscopy

Circular dichroism spectra were recorded on a Jasco J-815 spectropolarimeter. All spectra were recorded with 1 nm steps and a dwell time of 2 s per step, using a 0.2 mm quartz cell (SUPRASIL, Hellma GmbH, Germany), in the wavelength range of 180-260 nm. Protein concentration was 25 μM.

^{199m}Hg perturbed angular correlation spectroscopy

^{199m}Hg PAC measurements were performed at ISOLDE beam line at CERN. Radioactive mercury was generated by irradiating a liquid Pb target with protons (1 GeV). ^{199m}Hg isotopes were selected with an on-line mass separator, and the ion beam was collected into ca. 150 μL ice kept in a Teflon holder cooled by a cold finger.

The radioactive ^{199m}Hg solution was mixed with non-radioactive $HgCl_2$ solution in Tris/$HClO_4$ buffer (pH = 7.5), followed by the addition of the Wild-type or truncated CueR protein (in 20 mM Tris/$HClO_4$, 1 mM TCEP, pH 7.5). As the next step P*copA* dsDNA (in 20 mM Tris/$HClO_4$, 0.1 mM $NaClO_4$, pH 8.0) was added. The pH was adjusted to 7.46 (at 25 °C) or 5.73 (at 25 °C) by adding Tris or MES buffers and solutions of NaOH or $HClO_4$. Finally, sucrose (55 % *w/w*) was added to the mixtures and dissolved. The data were recorded on a 6-detector (BaF_2) instrument at 1 °C. The pH values of samples were verified after the measurements and the decay of radioactive mercury.

The fitting was carried out using 300 data points (except the first 10 due to systematic errors in these), using a Lorentzian line shape for the static line broadening. The background subtraction in each of the coincidence groups was estimated using channels 550-600 (i.e. ~12 lifetimes of the intermediate nuclear level after t_0). t_0 for each group was determined using data from an experiment at the beginning of the beam time and at the end of the beam time, and gave values which were all the same within one channel (0.05 ns). Fourier transformation of the data and fits were carried out using 300 data points and a Keiser-Bessel window with the window parameter equal to 4. The time resolution and the time per channel were 0.52 ns and 0.05019 ns, respectively. Certain coincidence groups in the spectra of WT CueR (11, 17, and 23; all of which have detector 5 providing the stop pulse) exhibited systematic deviation (different from that originating from NQIs, and visible as a "bump" ~100 channels after t_0) from the exponential decay of the intermediate nuclear level, and these groups were not included in data analysis.

Quality control of the CueR proteins

The seven amino acid long *C*-terminal segment (starting from G128) was deleted in the truncated variant of CueR, hereafter denoted ΔC7-CueR. This mutant mimics the coordination properties of the metal ion binding loop of CueR displaying the residues C112 and C120 thus, comparison of the metal ion binding features of this protein with the Wild-type CueR may help to reveal a possible function of the CCHH motif. The integrity of the purified ΔC7-CueR was analysed by SDS-PAGE (Figure S1), and mass spectrometry (Figure S2). The deconvoluted mass spectrum indicated a 14462.29 Da monoisotopic molecular mass for the singly charged intact protein (MH^+) which corresponds well to the calculated value (14462.31 Da). The similarity of the circular dichroism spectra of the truncated and WT CueR (Figure S3A) suggested that the modification did not change significantly the secondary structure of the

protein. Moreover, electrophoretic mobility shift assay (EMSA) revealed that the DNA binding specificity of the truncated protein was maintained (Figure S3B).

^{199m}Hg PAC measurements with WT CueR in the presence of DNA

At pH 8.0 and in the absence of DNA, the picture is qualitatively similar to that observed in the presence of DNA (Figure S7). Clearly, there are no major differences induced by the binding of DNA, and accordingly, the coordination number of Hg^{II} does not change. Consequently, the spectrum recorded with 0.2 equivalent Hg^{II} without DNA at pH 8 may be interpreted in terms of structures as indicated in the main text, *i.e.* an equilibrium between HgS_2 and T-shaped HgS_3 coordination geometries. It is possible that the equilibrium between the two species observed at 0.2 equivalent Hg^{II} is shifted slightly towards the low frequency component, which presumably reflects the HgS_3 structure, but the accuracy of the fitted amplitudes is not adequate to make an indisputable conclusion on this issue. The spectra recorded with 1.0 equivalent Hg^{II} with and without DNA present are highly similar, exhibit subtle differences, mainly a slightly higher frequency and lower asymmetry parameter in the presence of DNA. This appears to be significant, as the two first peaks are more separated in the presence of DNA than in the absence of DNA. This might be due to a purer linear HgS_2 coordination geometry in the presence of DNA, possibly reflecting that the coordinating donor groups in the equatorial plane are farther away from Hg^{II}.

Model for the binding of Hg^{II} to the functional and *C*-terminal binding sites

For each protein dimer there are 4 metal binding sites, and they come in pairs: M1 and M2 at one end, and M1´ and M2´ at the other end. It is reasonable to assume that if both M1 and M2 are occupied by Hg^{II} both will be HgS_2, and there will be no HgS_3 coordination geometries. In addition, the affinity of the protein for Hg^{II} is so high that free Hg^{II} may be neglected.

Make the following assumptions:

1) Hg^{II} is randomly distributed among the metal sites, *i.e.* they are independent sites
2) If only M1 or M2 is occupied HgS_3 may be formed, because the free thiols of the adjacent site are available for coordination. The equilibrium between HgS_2 and HgS_3 may be estimated from the relative population derived from the amplitudes of the two

species in the ^{199m}Hg PAC data with 0.2 eq. Hg^{II} at pH 8, in the absence of DNA, $[HgS_2]/[HgS_3]$ = 1.5±1.1, see Table S1.

With these assumptions, we can compare the ^{199m}Hg PAC data with the UV absorption spectra at any Hg^{II}/CueR ratio, as exemplified in the following for the experiment performed with 1.0 eq. Hg^{II} per protein.

With 1.0 eq. Hg^{II} the probability of M1 being occupied is 0.5 (and correspondingly the probability of being vacant is 0.5). Thus, the probability of M1 and M2 being occupied at the same time is 0.5*0.5, and thus the probability of HgS_2 due to both sites being occupied is 0.25 (since there are two sites it is multiplied by 2 in the following). The probability of M1 being occupied and M2 not being occupied is 0.5*0.5, and vice versa (M2 occupied and M1 not being occupied is also 0.5*0.5), in total 2*0.5*0.5 = 0.5. In this case we know $[HgS_2]/[HgS_3]$ = 1.5±1.1, so out of this 0.5, 0.3 gives HgS_2 and 0.2 gives HgS_3. Thus in total HgS_2/HgS_3 = (2*0.25+0.3)/0.2 = 4 (in fact, given the standard deviation of 1.1, HgS_2/HgS_3 is predicted to be within the range from 1.8-5.5 at 1 eq. Hg^{II} per protein) in agreement with the UV data giving HgS_2/HgS_3 ~ 2.5, using the data in Figure 4A and 4C.

Figures

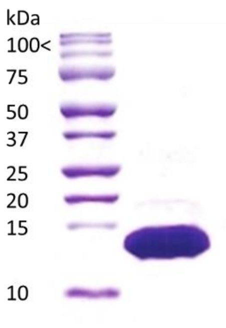

Figure S1: SDS-PAGE analysis of the purified ΔC7-CueR protein.

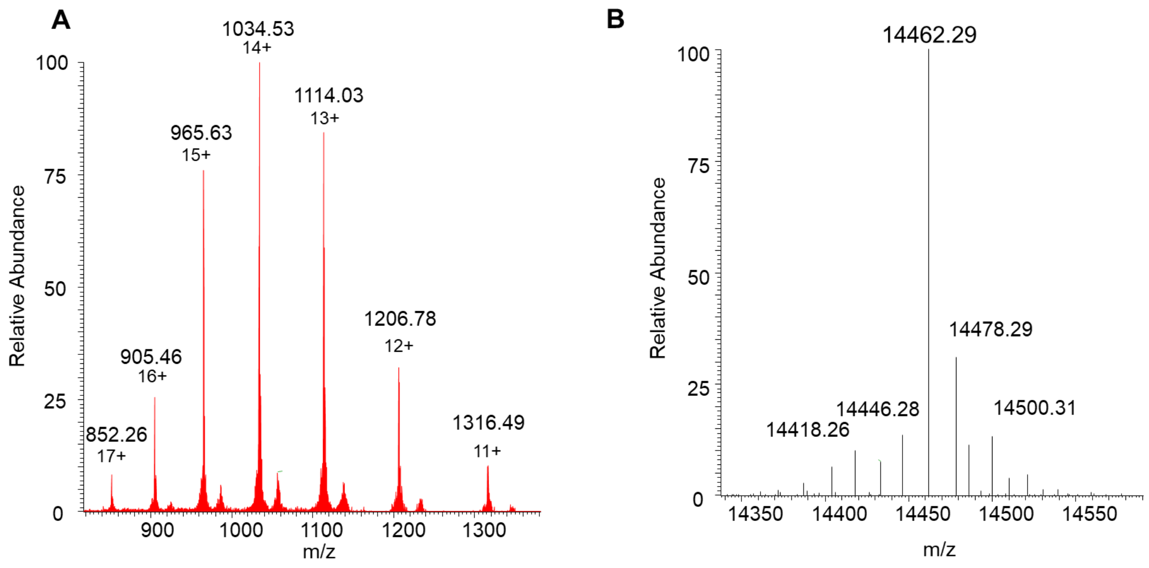

Figure S2: Measured (A) and deconvoluted monoisotopic mass spectra (B) of the purified ΔC7-CueR protein.

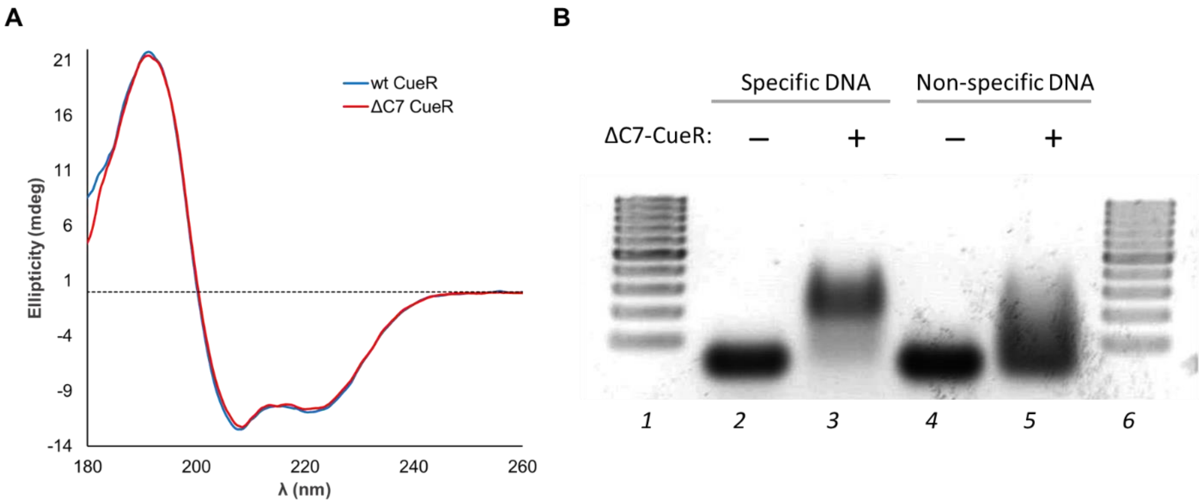

Figure S3: (A) CD spectra of the Wild-type and ΔC7-CueR proteins (c_{protein} = 25 μM) **(B)** EMSA analysis of ΔC7-CueR and 35 bp specific and non-specific DNA fragments. Lane *1* and *6* contain the 100 bp DNA ladder (GeneRuler, Thermo) for comparison. The protein-free specific and non-specific DNA samples are in lanes *2* and *4* respectively, while protein-DNA mixtures are in lanes *3* and *6*.

Apo-CueR | Hg-CueR | Hg_2-CueR

Exp.

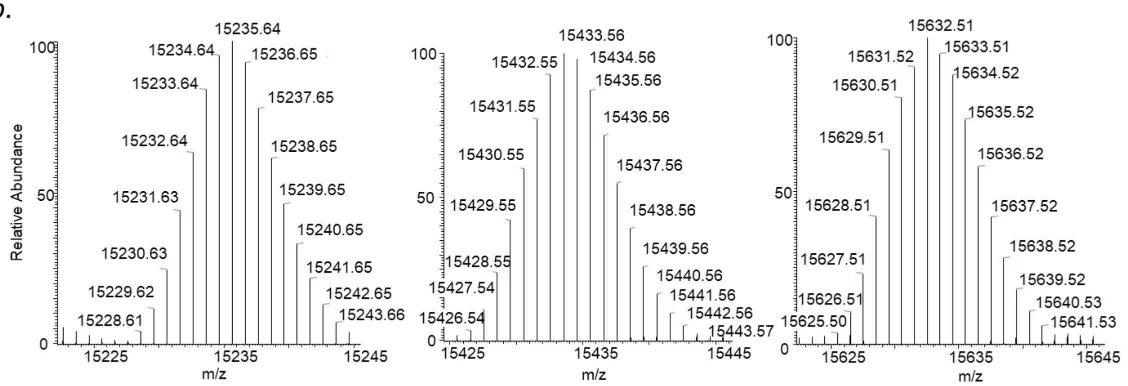

Calc.

Elemental Composition: **C652 H1055 N198 O209 S7**

m/z	%
15226.60516	0.10
15227.60800	0.84
15228.61074	3.58
15229.61338	10.31
15230.61594	22.67
15231.61842	40.57
15232.62085	61.47
15233.62321	81.04
15234.62552	94.83
15235.62778	100.00
15236.63000	96.15
15237.63219	85.11
15238.63434	69.88
15239.63646	53.58
15240.63855	38.56
15241.64062	26.18
15242.64267	16.83
15243.64471	10.27
15244.64674	5.97
15245.64878	3.32
15246.65083	1.76
15247.65289	0.89
15248.65501	0.42
15249.65728	0.19
15250.65950	0.08
15251.66140	0.03
15252.66337	0.01
15253.66541	0.00

C652 H1052 N198 O209 S7 Hg1

m/z	%
15419.54750	0.00
15420.55029	0.00
15421.55008	0.02
15422.55168	0.13
15423.55379	0.62
15424.55600	2.13
15425.55819	5.67
15426.56033	12.43
15427.56242	23.28
15428.56448	38.19
15429.56650	55.81
15430.56851	73.63
15431.57051	88.54
15432.57250	97.83
15433.57449	100.00
15434.57648	95.07
15435.57845	84.49
15436.58043	70.49
15437.58240	55.41
15438.58436	41.17
15439.58633	29.00
15440.58829	19.42
15441.59025	12.38
15442.59222	7.52
15443.59420	4.36
15444.59620	2.40
15445.59822	1.25
15446.60029	0.62
15447.60236	0.28
15448.60444	0.11
15449.60639	0.04
15450.60850	0.01
15451.60926	0.00

C652 H1051 N198 O209 S7 Hg2

m/z	%
15617.50979	0.00
15618.50994	0.00
15619.51114	0.02
15620.51279	0.11
15621.51466	0.40
15622.51658	1.24
15623.51849	3.15
15624.52038	6.91
15625.52223	13.33
15626.52407	23.01
15627.52589	35.96
15628.52770	51.40
15629.52951	67.69
15630.53132	82.63
15631.53313	93.98
15632.53495	100.00
15633.53677	99.92
15634.53860	94.04
15635.54043	83.62
15636.54228	70.42
15637.54413	56.28
15638.54599	42.77
15639.54785	30.97
15640.54973	21.39
15641.55161	14.10
15642.55350	8.86
15643.55540	5.32
15644.55733	3.03
15645.55927	1.64
15646.56122	0.83
15647.56319	0.39
15648.56518	0.17
15649.56700	0.06
15650.56912	0.02
15651.56983	0.00
15652.57632	0.00

Figure S4: Comparison of the experimental and calculated isotopic envelop of WT CueR, Hg-WT CueR and Hg_2-WT CueR species (calculations were executed with Protein Prospector ver. 5.22.1 – UCSF).

Apo-ΔC7CueR Hg-ΔC7CueR

Exp.

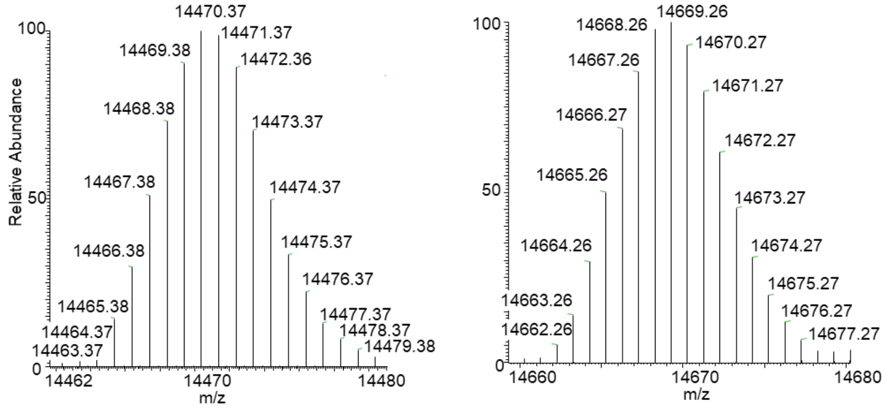

Calc.

Elemental Composition:

C623 H1011 N184 O202 S5

m/z	%
14462.30928	0.16
14463.31214	1.29
14464.31491	5.20
14465.31760	14.23
14466.32023	29.70
14467.32279	50.39
14468.32529	72.34
14469.32774	90.30
14470.33015	100.00
14471.33251	99.74
14472.33484	90.67
14473.33714	75.83
14474.33941	58.82
14475.34165	42.58
14476.34387	28.93
14477.34607	18.53
14478.34826	11.23
14479.35044	6.46
14480.35261	3.54
14481.35481	1.85
14482.35702	0.92
14483.35928	0.43
14484.36164	0.19
14485.36411	0.08
14486.36650	0.03
14487.36930	0.01
14488.37067	0.00

C623 H1009 N184 O202 S5 Hg1

m/z	%
14656.25945	0.00
14657.26224	0.00
14658.26194	0.03
14659.26358	0.20
14660.26573	0.91
14661.26797	3.00
14662.27019	7.69
14663.27236	16.22
14664.27448	29.22
14665.27658	46.06
14666.27865	64.67
14667.28072	81.90
14668.28279	94.46
14669.28485	100.00
14670.28692	97.82
14671.28900	88.91
14672.29107	75.45
14673.29315	60.04
14674.29522	44.98
14675.29730	31.81
14676.29938	21.32
14677.30147	13.56
14678.30356	8.20
14679.30566	4.72
14680.30779	2.58
14681.30991	1.34
14682.31208	0.66
14683.31434	0.30
14684.31650	0.12
14685.31885	0.04
14686.32123	0.01
14687.32309	0.00

Figure S5: Comparison of the experimental and calculated isotopic envelop of ΔC7-CueR and Hg-ΔC7-CueR species (calculations were executed with Protein Prospector ver. 5.22.1 – UCSF).

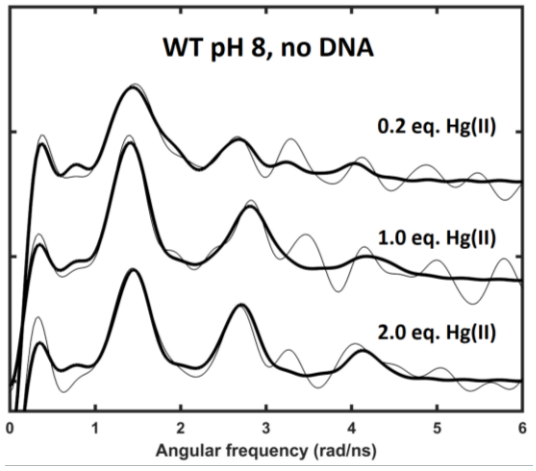

Figure S6: Experimental (grey) and fitted (black) ^{199m}Hg PAC spectra of the WT CueR in the absence of DNA at pH = 8.0 with 0.2, 1.0 and 2.0 equivalents of Hg^{II}. $c_{\mathrm{WT\ CueR}}$ = 11.5 μM.

Tables

Table S1 Parameters fitted to the ^{199m}Hg PAC-data for the WT CueR, see Hemmingsen et al. 2004[4] for a description of the parameters. The numbers in parenthesis are the standard deviations of the fitted parameters a: Can be analysed with only one NQI, but then the fit gives either too low amplitude (high eta, and the fact that the third peak appears to rise above the noise indicates that the high eta NQI alone is not adequate to achieve a good fit) or too high amplitude (low eta). b: faster reorientation may reflect less rigid geometry at site 2. c: It is conceivable that this relatively high value (as compared to the other datasets) may reflect the presence of another NQI (as observed with 0.2 eq. Hg^{II}), and indeed the fit is not as satisfactory, but the reduced chi-square is not statistically significantly improved by including a second NQI, and therefore, only one NQI is used to fit the data. d: Fast reorientation may reflect exchange between the two species. e: The presence of DNA bound to CueR does not give rise to a measurable change in $1/\tau_c$.

Hg^{II} : WT CueR	$c_{\mathrm{protein,final}}$	$c_{\mathrm{Hg'',final}}$	$c_{\mathrm{DNA,final}}$	pH	ν_Q	η	δ	$1/\tau_c$	A	χ_r^2
	(μM)	(μM)	(μM)		(GHz)		×100	μs^{-1}	×100	
0.2	11.5	2.30	5.68	6.0	1.48(1)	0.13(4)	0(1)	7(3)	13(2)	0.82
0.2[a]	11.5	2.30	5.68	8.0	1.48(1)<br>1.13(2)	0(1)<br>0.56(8)	0(2)<br>0(4)	16(3)[d]	9(2)<br>5(1)	0.78
1.0	11.5	11.5	5.68	6.0	1.474(9)	0.18(2)	0(1)	8(2)	13(1)	0.66
1.0	11.5	11.5	5.68	8.0	1.49(2)	0.23(4)[c]	2(2)	8(5)	12(2)	0.71
2.0	11.5	23.0	5.68	6.0	1.49(1)	0.12(5)	0(2)	17(4)[b]	15(1)	0.75
2.0	11.5	23.0	5.68	8.0	1.53(1)	0.15(5)	2(2)	10(3)[b]	14(2)	0.67
0.2[a]	11.5	2.30	0	8.0	1.42(2)<br>1.04(4)	0.1(1)<br>0.77(9)	0(3)<br>0(6)	11(4)[d]	6(3)<br>4(2)	0.71
1.0	11.5	11.5	0	8.0	1.49(2)	0(1)	5(2)	7(2)[e]	16(2)	0.75
2.0	11.5	23.0	0	8.0	1.45(1)	0.23(3)	1(2)	12(4)[b]	13(2)	0.80

Table S2: Parameters fitted to the ^{199m}Hg PAC-data for ΔC7-CueR with the same sample conditions.

Hg^{II} : ΔC7-CueR	$c_{protein,final}$	$c_{Hg^{II},final}$	$c_{DNA,final}$	pH	ν_Q	η	δ	$1/\tau_c$	A	χ_r^2
	(μM)	(μM)	(μM)		(GHz)		×100	us^{-1}	×100	
0.2	8.45	1.69	4.23	6.0	1.55(2)	0.07(9)	8(2)	23(17)	15(2)	0.62
0.2	8.45	1.69	4.23	8.0	1.55(1)	0.16(3)	3(2)	120(27)	14(1)	0.66
1.0	8.45	8.45	4.23	6.0	1.55(2)	0.13(5)	6(2)	78(26)	15(2)	0.72
1.0	8.45	8.45	4.23	8.0	1.52(2)	0.16(5)	6(2)	16(22)	12(2)	0.75
2.0	8.45	16.9	4.23	8.0	1.60(4)	0.15(7)	10(3)	97(37)	16(2)	0.56

References

[1] F. W. Outten, C. E. Outten, J. Hale, T. V. O'Halloran, *Journal of Biological Chemistry* **2000**, *275*, 31024-31029.

[2] R. K. Balogh, B. Gyurcsik, É. Hunyadi-Gulyás, H. E. M. Christensen, A. Jancsó, *Protein Expression and Purification* **2016**, *123*, 90-96.

[3] H. Schägger, *Nature Protocols* **2006**, *1*, 16.

[4] L. Hemmingsen, K. N. Sas, E. Danielsen, *Chemical Reviews* **2004**, *104*, 4027-4062.
